# New Appliances and Things Medical

**Published:** 1905-05-27

**Authors:** 


### NEW APPLIANCES AND THINGS MEDICAL.

[We shall be glad to receive, at our Office, 28 & 29 Southampton Street, Strand, London, W.C., from the manufacturers, specimens of all new preparations and appliances which may be brought out from time to time.]

### A NEW POISON BOTTLE.

(The Locket Patents Co., 6 Mornington Place, Hampstead Road, N.W.)

Numerous attempts have been made to safeguard the public against the administration of a poison in mistake for a harmless drug. So far the devices have not proved infallible. The bottle sent for our inspection by the Locket Patents Company makes the unintentional use of the contents impossible. On the blue glass of the bottle a red label is fixed marked poison. On the label there is space for directions. The bottle has a cork or a glass stopper, over which is a screw metal cover which must always be replaced, as on this the bottle stands, the other end being convex. Even if the metal cover were omitted and the bottle left on its side, the destinction is sufficient to render mistake impossible. The contrivance is certainly simple and effective.

